# Characterization of Corn Silk Extract Using HPLC/HRMS/MS Analyses and Bioinformatic Data Processing

**DOI:** 10.3390/plants12040721

**Published:** 2023-02-06

**Authors:** Laëtitia Fougère, Sandrine Zubrzycki, Claire Elfakir, Emilie Destandau

**Affiliations:** Institut de Chimie Organique et Analytique, Université d’Orléans, CNRS, UMR 7311, F-45067 Orléans, France

**Keywords:** Kendrick and van Krevelen plot, molecular networking, UHPLC/HRMS, maysin and derivatives, flavonoids

## Abstract

In addition to having different biological activities of interest, corn silks play a role in the defense of plants. While benzoxamines and flavonoids have already been identified as molecules of plant defense and growth mechanisms, knowledge on the phytochemical composition of corn silk is lacking. Such knowledge would make it possible to better select the most effective varieties to improve resistance or bioactive properties. In this article, an approach was implemented to map a corn silk extract in two complementary ways. The first one involved working with UHPLC/HRMS data and Kendrick and van Krevelen plots to highlight a homologous series of compounds, such as lipids from 17 to 23 carbons, monoglycosylated flavonoids from 21 to 24 carbons, diglycosylated flavonoids of 26 to 28 carbons and organic acids of 14 to 19 carbons. The second way was to analyze the sample in UHPLC/HRMS^2^ and to plot mass spectral similarity networks with the GNPS platform and Cytoscape software to refine identification. By combining the information obtained, we were able to propose an identification for 104 detected molecules, including 7 nitrogenous, 28 lipidic and 67 phenolic compounds, leading to the first detailed phytochemical analysis of corn silk extract.

## 1. Introduction

Corn silks are used medicinally as a decoction, an infusion or in tablet form for a variety of applications. Traditionally, corn silks have been used for the treatment of urine disorders, swelling, asthma and hypertension [1]. They have shown various properties of interest such as antidiabetic [2], anticoagulant [3], antifungal [4] and antioxidant [5,6] activities, and as inhibitors of IgE antibodies by glycoproteins in the context of allergic diseases [7]. The composition of corn silk extract, which depends on the variety, determines its biological activity [8,9]. The polysaccharides contained in corn silk may have an antidepressant activity. The amount of phenolic compounds, such as flavonoids, determines the effectiveness of the antioxidant effect or the protective properties against atherogenesis.

Corn silk is an excellent source of bioactive compounds such as flavonoids, saponins, alkaloids, tannins, phytosterols, terpenoids, steroids, saccharides, cerebrosides, allantoin and vitamins E and K [10]. However, little is known about the chemical constituents of each molecular family. Only a few isolated flavonoids [9,11,12,13,14,15], some separated phenolic compounds [16] and benzoxamines have been reported [17]. These specialized metabolites are of great interest for their bioactivity or ecological roles, notably in plant defense, as benzoxamines have activity on plant growth or plant defense and flavonoids are well known to contribute to plant resistance [17,18,19]. Studies also indicate the involvement of flavonoids contained in maize silks in plant defense [20]. A few C-glycosylated flavonoids, among them maysin, have been isolated and have been shown to inhibit the growth of *Helicoverpa zea* larvae [21]. In addition, it was observed that while the defense efficiency of corn silk depends on the variety, it could not be explained solely by the composition or the amount of C-glycosylated flavonoids [22,23]. Therefore, a better knowledge of the phytochemical profile of corn silks would provide a better understanding of the plant defense mechanisms. Additionally, corn silk is a food by-product [24]. Several studies showed the use of corn silk in various food formulations, such as in meat balls [25]. However, it is conventionally used as manure or animal feed due to the lack of knowledge. Characterization of the bioactive compounds of this food by-product can help in further exploitation of corn silk.

To obtain an overall characterization of an extract, coupling between liquid chromatography and mass spectrometry is increasingly used. High-resolution mass spectrometry provides precise mass information, thus assisting in the identification of unknown compounds. In recent years, support for chemical analyses and mass spectrometry data processing has been developed through bioinformatics. Tools and web-platforms have multiplied to help with the study of metabolites and their identification. In 2016, Rathahao-Paris et al. reviewed the interest of working in high resolution mass spectrometry to identify metabolites [26]. They also showed the interest of mathematical tools, such as the mass defect and the van Krevelen diagram, in helping to visualize and characterize metabolites.

Given that in LC-MS^2^ it is not possible to build a global database for all types of technologies, a group of researchers has created a platform to display fragmentation similarities between metabolites. This method is called molecular networking (MN) [27]. Metabolites fragmenting in the same way are grouped together, so the compounds belonging to the same molecular families should be grouped in the same cluster. MN makes it possible to confirm the presence of known compounds and to determine the unknown compounds located in the same cluster by studying, step by step, the difference of exact masses [28].

The aim of this work was to perform a non-targeted characterization of a corn silk extract by combining the results obtained with different MS data-processing tools in order to map and characterize the different families of specialized metabolites contained in the extract. To refine the compound identification, UHPLC/HRMS-MS and molecular networking were then undertaken to obtain a first more detailed characterization of the flavonoids constituting the main compound family of the extract. To our knowledge, this study is the first to present a global characterization of a corn silk extract.

## 2. Results

**Figure 1:**
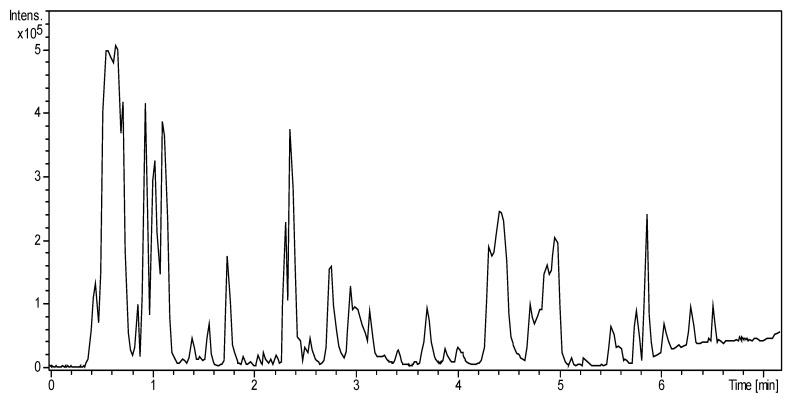
Base peak chromatogram in negative electrospray ionization of corn silk extract.

The base peak chromatogram of the analysis by UHPLC/HRMS of the corn silk extract is presented in Figure 1. An intense group of molecules is located close to the dead volume, which should be polar primary metabolites not retained on the C18 column. The other molecules come out depending on the polarity range from 20% to 100% acetonitrile. The literature allowed the assumption of the elution of molecular families such as phenolic and benzoxamine compounds at the middle of the gradient and of lipid compounds at the end. Around 200 compounds have been detected, and their exact mass and their molecular formula were determined to allow the construction of the Kendrick, van Krevelen and double bond equivalent diagrams.

### 2.1. MS Mapping

In the petroleum field, the characterization of complex products is frequently performed by the visualization and interpretation of Kendrick plots and van Krevelen diagrams. These methods have also been applied to different food matrices, such as tea [29] or wine [30].

The Kendrick diagram is represented by the Kendrick mass defect (KMD, i.e., the difference between nominal mass and Kendrick mass) as a function of mass [31]. Molecules that differ only by one or more alkyl groups will have the same mass defect. This makes it possible to differentiate between homologous series. Watrous et al. (2019) used Kendrick’s plot to characterize unknown eicosanoids using a difference in oxygen atoms [32]. Lipids with a mass below 400 Da have a small KMD, while for flavonoids, the more they increase in mass, the more their KMD will increase linearly.

The van Krevelen diagram gives a qualitative graphic representation of the distribution of the molecules according to the H/C and O/C ratios. The H/C ratio separates the compounds according to their degree of unsaturation, while the O/C ratio distinguishes the compounds according to the number of oxygen atoms, and the diagonals distinguish molecules with respect to their methylation and their hydration [33].

Molecular families of natural compounds possess different characteristic ratios, presented in Table 1, which make it possible to define specific zones in the van Krevelen diagram. The diagram can then be used to quickly determine whether the compounds of an unknown complex mixture belong to the different molecular families according to their crude formula (Table 1).

**Figure 2:**
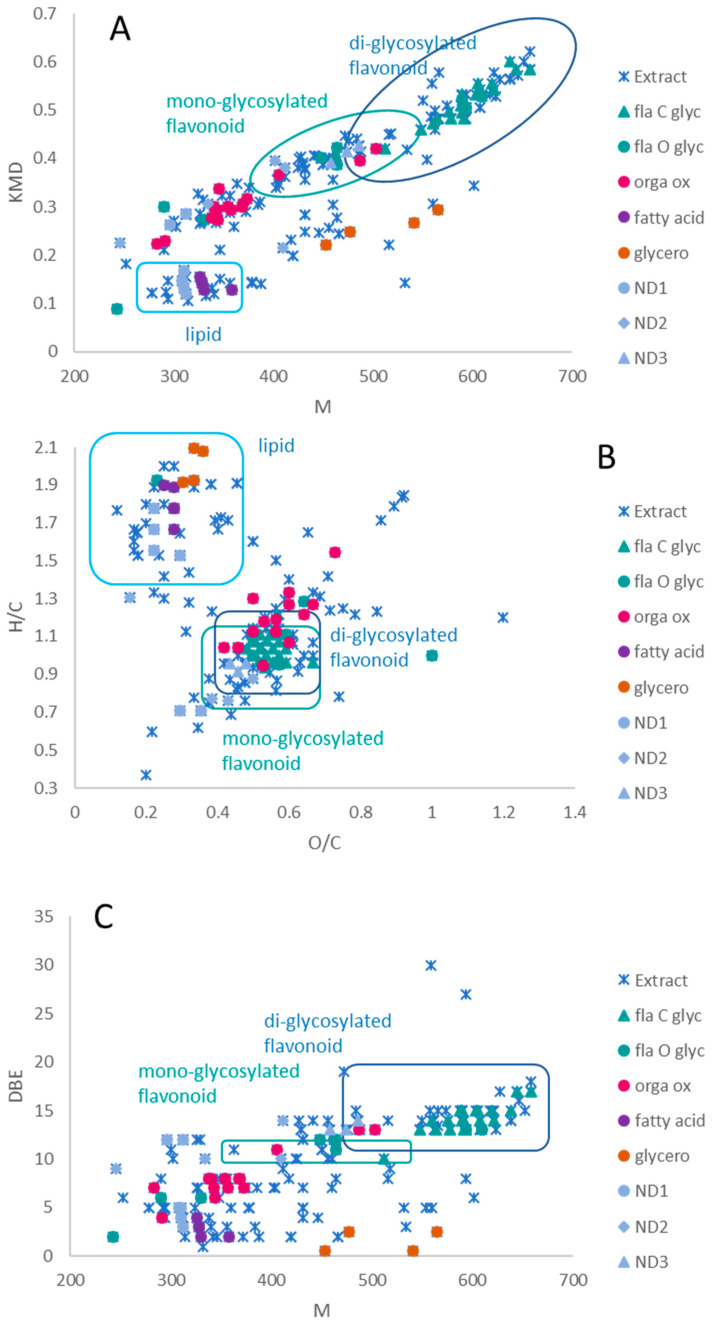
(**A**) Two-dimensional Kendrick molecular mass plotted against the Kendrick mass defect (KMD); (**B**) van Krevelen diagram showing the O/C ratio versus the H/C ratio; (**C**) double bond equivalent plot as a function of molecular weight. The blue crosses indicate the assigned molecule for corn silk extract analyzed in negative ionization mode. The ions clustered in the molecular network are labeled by the colored full forms, corresponding to molecular families (fla C glyc = flavonoid *C*-glycosylated; fla O glyc = flavonoid *O*-glycosylated; orga ox = organooxygen; fatty acid; glycero = glycerophospholipid; ND = not determined). The circled areas indicate the areas of the molecular families constructed from the Extrasynthese database.

Figure 2 shows the Kendrick (Figure 2A), van Krevelen (Figure 2B) and double bond equivalent (DBE) (Figure 2C) diagrams. They were constructed from the exact mass and molecular formula calculated from the pseudo-molecular ions ([M-H]^−^) detected in the corn silk extract. In order to define zones corresponding to the main molecular families of the extract on the diagrams, a database of natural compounds was extracted from the catalog of Extrasynthese (https://www.extrasynthese.com/ accessed on 1 December 2019) [36], and the areas corresponding to lipids, mono- and diglycosylated flavonoids have been circled on the diagrams. Among the molecules detected in the extract, compounds detected only with full scan MS are labelled with blue crosses, while the fragmented and clustered ions also found in the molecular network ions (cf. below) are highlighted on Figure 2 by colored circles according to the cluster of the molecular family. Diagrams based on full scan MS give a more exhaustive view of the chemical composition and a larger number of molecules detected compared to MS/MS analyses with MN.

Among the 200 ions of the extract, two distinct areas of molecules were identified with the Kendrick and van Krevelen diagrams. An area of about twenty molecules was observed with low masses, a low mass defect, little oxygen and a high H/C ratio, with a DBE of less than 5. Compounds inside this area correspond to lipids. A second zone of molecules was observed with a wide mass range, higher mass defects, but a lower H/C ratio, with DBEs between 10 at 15 and much more oxygen than the first group. This zone contains phenolic compounds. Those that have a higher molecular mass and higher H/C and O/C ratios correspond to diglycosylated molecules, while the others correspond to monoglycosylated compounds. In addition, by studying the molecular formulas of molecules, some alkaloid compounds were also detected. These compounds are in the same zone as the phenolic compounds, but were differentiated by the presence of nitrogen atoms. In what follows, we focus mainly on these two molecular families (lipids and phenolic compounds).

By combining the information from the different diagrams in Figure 2, a more detailed characterization of the 29 detected lipids can be proposed (Table 2). Figure 2A shows the series of homologs with differences in methyl function, taking into account the mass defect only, without considering the proposals for molecular formulas. Figure 2B made it possible to follow, step by step, the oxidations, hydrogenations, methylations, hydrations and combinations of these reactions. Lastly, the unsaturations are highlighted in Figure 2C. The lipid molecules are presented in Table 2, with the carbon number, the double bond and the oxygen number in the alkyl chain (without counting those of the acidic function).

Thus, the ions *m/z* 291.196 and 293.2117 (**compounds 7**; **9**), *m/z* 307.1911, 309.2068, 311.2222 and 313.2383 (**compounds 8**; **10**; **13**–**14**; **17**), and *m/z* 325.2014, 327.2165, 329.2327 and 331.2488 (**compounds 11**–**12**; **15**–**17**; **19**; **20**) show only a decrease in their degree of unsaturation. This means that these groups of ions belong to the same series of C18 homologs, with one, two and three hydroxyl functions, respectively. On the other hand, the ions *m/z* 309.1703 (**compound 5**) and 293.1754 (**compound 3**) have a different molecular formula, indicative of a different structure. This means that they belong to another series of C17 homologs with the *m/z* ions 277.1808 (**compounds 1**–**2**). These **compounds** (**1**–**5**) have a constant degree of unsaturation of five and a constant H/C ratio of 1.53, but with a hydroxyl function number which differs. A homologous C20 series is also present with the *m/z* 337.2374, 339.2532, 355.2484 and 357.2642 (**compounds 21**–**24**), which differ either by their degree of unsaturation or by their oxidation number. Lipids are mainly represented in this extract by three series of molecules C17, C18 and C20, with a different molecular formula. C23 (**compounds 25**–**29**) isomeric compounds were also detected.

The 67 phenolic compounds detected are listed in Table 3, classified by sugar number on the genin and by increasing carbon number; then, at the end of the table, the organic acids are listed by increasing carbon number. Table 3 also provides the name of the molecule if it has already been described, or at least the first description of the molecule that was implemented, by indicating the family of the genin with the oxygen number surrounding it and the sugar number. For the same molecular formula, a difference in oxygen number was sometimes observed, because the hydroxyl may sometimes be carried by the genin or the sugar. These annotations were refined based on bibliographic correspondence and the study of the fragmentation mass spectra obtained. With the information given by MS data, flavone and flavonol genins cannot be distinguished, nor can flavanone and flavanonol genins. The basic structure of a flavone has fifteen carbons and two oxygens, while a flavonol has the same number of carbons, but with one more hydroxyl. Therefore, a flavonol cannot be distinguished from a flavone with a hydroxyl. In Table 3, genins are classified as flavones by default, except when there is a more precise characterization, as flavones have been described more frequently in the literature on *Zea mays*. The same goes for flavanone and flavanonol with one less unsaturation. They differ only by one hydroxyl position, and thus cannot be distinguished under these analytical conditions. For some molecules in Table 3, no description is proposed, because there was too much doubt about the structure, which was not resolved by the fragmentation of the molecules.

The ions *m/z* 559.1453 (**compound 11**) and 575.1401 (**compound 12**) have already been described as apimaysin and maysin. These molecules are, respectively, an apigenin and a luteolin with a *C*-oxodeoxyhexose followed by an *O*-deoxyhexose. The molecules are made up of 27 carbons and 28 hydrogens, with a DBE of 14 and an H/C ratio of 1.037. In the same series, ions *m/z* 591.1344 (**compound 13**) and 607.1299 (**compounds 14**) were observed. These molecules can have the same structure as the first two, with one or two additional hydroxyl functions. With the same DBE of 14, the ion *m/z* 589.1559 (compound **28**) was observed, which corresponds to 3′-methoxymaysin. This molecule has an additional methoxy group compared to apimaysin. It has an H/C ratio of 1.071, 28 carbons and 30 hydrogens. In the same series, ions *m/z* 621.1459 (**compound 31**) and 637.1414 (**compounds 29**–**30**) were observed. They have three to four hydroxyl functions in addition to the 3′-methoxymaysin molecule. Other molecules have 27 carbons, close to the apimaysin molecule. The ions *m/z* 561.1614, 577.1559, 593.1511 and 609.146 (**compounds 15**–**23**) were observed. Compared to this first series, they have an H/C ratio of 1.111 and a DBE of 13, so one less unsaturation. Likewise, ions *m/z* 557.1299, 573.1248, 589.1208, 605.1151, 621.1088 and 637.104 (**compounds 4**–**10**) have an H/C ratio of 0.963 and a DBE of 15; they therefore have an additional unsaturation compared to the first series. Similarly, for the C28 series, ions *m/z* 587.1399, 603.135 and 619.13 (**compounds 24**–**26**) and ions *m/z* 591.1723, 607.1663 and 623.1612 (**compounds 32**–**35**) were observed. They correspond, respectively, to one more unsaturation, with a DBE of 15 and an H/C ratio of 1, and to one less unsaturation, with a DBE of 13 and an H/C ratio of 1.143. Two other series were observed in C26 and C30, which are combinations of methoxy, hydroxyl and unsaturation functions more or less comparable to the C27 and C28 series. This approach made it possible to characterize the majority of molecules located between 500 and 680 as being diglycosylated flavonoids. Most of the C21, C22, C23 and C24 molecules can be explained by the loss of a sugar (hexose, deoxyhexose or pentose) compared to the diglycosylated molecules previously described.

Molecules with a molecular mass less than 400 Da can correspond either to aglycon flavonoids or to organic acids. From the literature, ion *m/z* 353.0877 (**compound 62**) has already been described as chlorogenic acid. Organic acids are eluted at low retention times (<3 min). C16 (**compounds 59**–**61** and **compounds 63**–**64**) (*m/z* 337.0926, 355.1031) have been described as analogs of chlorogenic acid with one hydroxyl function or one unsaturation less. The C15 (**compounds 55**–**57**) (*m/z* 343.1035, 339.0721, 325.0922) and C17 (**compounds 65**–**67**) (*m/z* 367.1033, 405.0797) series, due to the addition or removal of a methyl function, have been described as organic acids.

To refine the identification proposed from the Kendrick, van Krevelen and DBE diagrams, the exact mass and molecular formulae obtained were compared with the molecules described in the literature of *Zea mays* and listed in the lotus database (https://lotus.naturalproducts.net/ accessed on 7 August 2022) [37]. This comparison confirms the propositions of molecular formulas of the concordant molecules and enables dereplication. Thus, this corn silk extract contains 14 known phenolic compounds which have already been described in the literature (indicated by an asterisk * in Table 3). This method also highlighted numerous undescribed molecules. Thus, in order to characterize them, a data dependent acquisition was carried out to obtain fragmentation spectra.

### 2.2. Mass Spectral Similarity Networking

If the analyses are carried out with fragmentation, a representation by spectral similarity can be used. The principle is the grouping of molecules according to their common fragments. Consequently, a cluster groups molecules with the same fragmentation pathway which should therefore belong to the same molecular family. A recent study on maize leaves used molecular networks to highlight the impact of biostimulants on the metabolism of maize plants under normal and drought conditions [38].

**Figure 3:**
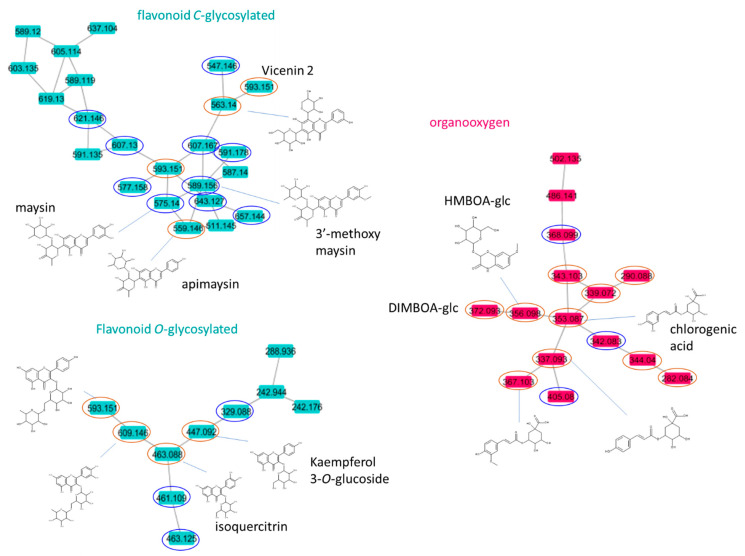

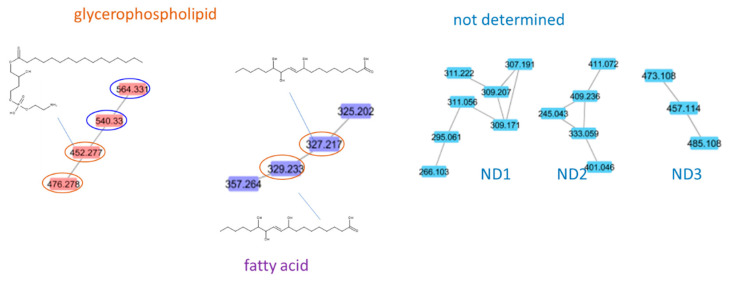
Representation of molecular networking applied to UHPLC/QT of MS/MS data from corn silk extract using electrospray ionization in the negative mode, with coloration based on the chemical class (MS2LDA), and identification of certain nodes via the GNPS library (red circles) and the proposition of structures (blue circles).

The molecular network of corn silk extract consists of a total of 141 nodes. It exhibits eight main clusters formed with 79 ions in the negative ionization mode, presented in Figure 3. This ionization mode showed more clustered ions in comparison to the positive ionization mode (figure not shown), and thus is better adapted to describe the extract composition. The compound family identification using MS2LDA showed that the network consisted of two clusters identified in the family of phenylpropanoids or polyketides, a cluster of so-called organooxygen compounds, two clusters of lipids and three undetermined clusters. The search in the GNPS library resulted in a match with 24 compounds (red circles on Figure 3). Among these compounds, the library identified eight flavonoids and seven organooxygen compounds, which correspond to phenolic acids or benzoxazinoids, three other nitrogenous compounds (amino acid or nucleotides), two fatty acids and four glycerophospholipids. These molecular families are found in the literature on *Zea mays*, except for the family of glycerophospholipids. The comparison of the GNPS library hits with the molecules already described in *Zea mays* confirms the presence of seven molecules in our extract: isoquercitrin *m/z* 463.088 (**compound 44**), kaempferol-3-*O*-glucoside *m/z* 447.092 (**compound 43**), apimaysin *m/z* 559.146 (**compound 11**), vicenin 2 *m/z* 593.151 (**compound 19**), DIMBOA-glc *m/z* 372.093, HMBOA-glc *m/z* 356.096, and guanosine *m/z* 282.084 (not listed in Table 2 or Table 3, but annotated in MN in the organooxygen cluster on Figure 3).

The putative identifications in Table 4 are the identifications in accordance with GNPS and the literature, and with a match of the fragmentation spectra. When there was no match, propositions were made either by de novo interpretation of the MS^2^ spectra, or by a description via the diagrams.

## 3. Discussion

Nurraihana et al. (2018) carried out a first identification of 21 flavonoids of corn silk extract by LC/MS [39]. Some molecules characterized have common molecular formulas with those of this analysis; however, MS data led to the interpretation of different structures. For example, the molecular formula C_26_H_28_O_13_ was characterized in their extract as being the molecule mirificin, which corresponds to an isoflavone, daidzein 8-*C*-glucoside 2″-*O*-apioside. In comparison, in this extract it was characterized as a flavone, apigenin 6-*C*-deoxyhexose 8-*C*-pentose. The compound characterization remains the main challenge, because of the lack of a universal and complete database, due to the fact that the fragmentation spectra depend on the conditions of analysis and equipment. This is why Desmet et al. applied a novel approach of working with the candidate substrate–product pair (CSPP) networks by combining them with spectral metadata in different organs of maize, which allowed them to perform structural characterization of 427 compounds out of the 5420 profiled compounds [40]. In this same way, this work combined different diagrams with molecular networks. Wolfender et al. (2019) showed that molecular networking can be used in the identification of polyphenols with library research and cross-checking information with retention time and spectrum comparison in silico [41]. Pilon et al. (2019) showed that the *O*-glycosylated flavonoid compounds can be well characterized with molecular networking [42]. Kouamé et al. (2021) used a molecular network approach to cluster *C*-glycosylated flavones and annotate them [43].

Here, with a cosine score value of 0.6, the two phenylpropanoid clusters were distinguished into a flavonoid *O*-glycosylated cluster and a flavonoid *C*-glycosylated cluster (Figure 3) since they present different fragmentation pathways. For O-glycosylated molecules, the main fragmentation occurs between the genin and sugar substituent, while for C-glycosylated flavonoids, fragmentation takes place inside the sugar substituent. The fragments of the *O*-glycosylated flavonoids are 162, 146 and 132 Da for hexose, deoxyhexose and pentose sugars, while when these sugars are bound in the C position, they give fragments of 120, 104 and 90, respectively. Clusters can be used to distinguish subfamilies of flavonoids according to *O-* or *C*-glycosylation. However, mono- and di-*O*-glycosylated flavonoids were grouped in the same cluster since they give the same genin fragment due to sugar loss and due to RDA rearrangement.

Some masses of molecules described in the literature were found in the molecular network, including the three most widely described flavonoids (maysin *m/z* 575.1401 (**compound 12**), apimaysin *m/z* 559.1453 (**compound 11**) and 3′-methoxymaysin *m/z* 589.1559 (**compound 28**)), which are found in the flavonoid *C*-glycosylated cluster. These three molecules differ in the presence of an additional hydroxyl function or an additional methyl function. The observation of the three mass spectra confirms the annotation of the three molecules. A loss of 102 and 164 is observed for these three molecules, losses that correspond, respectively, to the loss of an oxodeoxyhexose unit linked in C-C (^0,2^X_0_^−^) on the flavonoids and to the loss of a deoxyhexose linked in 2″-*O* on the latter (Z_1_^−^). The structure of *C*-[2″-*O*-glycosyl]-glycosides was evidenced by the presence of an [Ag + 41–18]^−^ product ion. Additionally, when searching the GNPS spectrum library, the apimaysin compound was found. In this same cluster, the GNPS library proposes three other identifications in *C*-glycosylated flavonoids. Another *C*-[*O*-glycosyl]-glycoside flavonoid is given as a flavone, with four oxygens and *C*-hexose-*O*-hexose, which could correspond to isoorientin 2″-*O*-rhamnoside (593.151 *m/z* (**compound 22**)). Two other molecules are proposed as di-*C*-glycosylated flavonoids, i.e., the molecules apigenin di-*C*-hexose (593.151 *m/z* (**compound 19**)) and isoshaftoside (563.14 *m/z* (**compound 3**)), as in the spectra of these two molecules a neutral loss corresponding to the break in ^0,2^X^−^ and ^0,3^X^−^, as well as the [Ag + 83]^−^ and [Ag + 113]^−^ product ions, were found. By propagation, six other molecules were annotated in this *C*-glycosylated cluster (621.1459 *m/z* (**compound 29**), 607.1664 *m/z* (**compound 33**), 607.1299 (**compound 14**), 591.1723 *m/z* (**compound 32**), 577.1552 *m/z* (**compound 18**) and 547.146 *m/z* (**compound 2**). These molecules differ from the previous ones either by a hydroxyl or ketone or methoxy function.

Similarly, two of the di-*O*-glycosylated flavonoid compounds (nicotiflorin *m/z* 593.1511 (**compound 21**), rutin *m/z* 609.146 (**compound 23**)) and two mono-*O*-glycosylated flavonoid compounds (kaempferol 3-*O*-glucoside *m/z* 447.0924 (**compound 43**), isoquercitrin *m/z* 463.0881 (**compound 44**)) were found in the GNPS library. Two of these proposals (rutin and nicotiflorin) from the GNPS library matched the molecular formulas in the literature, but the proposed name does not agree with the literature. The name of the molecule already described in the maize literature is reported in Table 4. By propagation, three other compounds of the *O*-glycosylated flavonoid cluster were described (461.109 *m/z* (**compound 49**), 463.125 *m/z* (**compound 50**) and 329.088 *m/z* (**compound 55**)). **Compound 49** differs from **compound 44** by one methoxy function, and compound **50** differs from c**ompound 49** by genin. **Compound 50** is therefore a flavanone, whereas **compound 49** is a flavone.

While Miao et al. (2020) determined seven classification groups (lipids, proteins, carbohydrates, unsatured hydrocarbons, lignins, tannins and condensed aromatics) by the van Krevelen diagram of root exudates [44], the corn silk extract diagrams highlighted three main areas of molecular families (lipids, organic acids, and mono- and diglycosylated flavonoids). To combine the information given by the different representations of the phytochemical composition of corn silk extract, the molecules clustered in the network are shown in the different diagrams (Figure 2). The two phenylpropanoid clusters are located in the flavonoid area. The cluster of *C*-glycosylated flavonoids contains more di-glycosylated flavonoids, while that of *O*-glycosylated flavonoids contains more mono-glycosylated flavonoids (Figure 2A). Based on Figure 2C, it can be stated that the compounds of the *C*-glycosylated flavonoid cluster have a DBE between 10 at 17. The DBE of 13 corresponds to the number of unsaturations of a flavone (11) with two unsaturations for sugars. Six compounds have a DBE at 14, of which the maysin (**compound 12**), apimaysin (**compound 11**) and 3′-methoxymaysin (**compound 28**) already described. These three molecules are flavones with two sugars. As one of the sugars has a ketone, they have one more unsaturation than diglycosylated flavones. The other three molecules (**compounds 29**–**31**) have a molecular formula similar to that of the 3′-methoxymaysin (C_28_H_30_O_14_) molecule with an additional oxygen, while the *O*-glycosylated flavonoid cluster has a DBE predominantly at 11 and 12. This confirms that they are monoglycosylated. In addition to monoglycosylated flavones, this group also contains a monoglycosylated flavanone (DBE 11), which has one less double bond, therefore one less unsaturation, similar to the compound hesperetin *O*-hexose (DBE 11).

In the organooxygen cluster, the GNPS library had more matches. Five phenolic molecules (coumarin and acids) (**compounds 56**, **58**, **60**, **62** and **65**), two benzoxazinoids and three other nitrogenous compounds (amino acids or nucleotides) are already listed in the library. The MS/MS spectra of these molecules showed common sugar neutral losses.

The molecules of the organooxygen compound cluster are more extensive in the representations (Figure 2A) due to mixtures of two main molecular families, organic acids and glycosylated benzoxazines. The glycosylated benzoxazines were found in different parts of the corn depending on the age of the plants [16], and they have a defense role during insect attack on maize plants [17]. The two families were distinguished in a van Krevelen diagram with the H/C axis versus the N/C axis.

Two other molecules were characterized by propagation, namely a phenolic acid (**compound 67**) and a benzoxazinoid.

Likewise, based on the correspondence with the GNPS library, the two clusters of lipids correspond to a cluster of glycerophospholipids and a cluster of fatty acids.

The two lipid clusters are not in the same place in the Kendrick diagram. The cluster of fatty acids is located in the lipid zone, whereas that of glycerophospholipids is located in an intermediate zone with a higher mass of lipids and a KMD lower than that of the mono-glycosylated flavonoids. The fatty acids have a DBE of less than 5 and the glycerophospholipids have a non-integer DBE value due to the presence of phosphorus. Two lipid clusters are well located in the lipid zone in the Kendrick diagram. The lipid clusters contain only eight compounds, while the lipid zone in Figure 2 contains around twenty compounds. The other lipids either fragmented with difficulty or did not have enough common fragments to bind to this cluster, such as molecules with an *m/z* of 327.217 (**compounds 15**–**17**), which are in the same node, and 329.2327 (**compound 19**). The molecular network does not give much information about the lipid composition and the best description is given by Table 1.

Concerning undetermined clusters, the largest cluster (ND1) is mostly in the lipid area, whereas the other two are in the phenolic compound area. The ND1 cluster could consist of fatty acids having 17 to 18 carbons, with 2 to 4 double bonds and 2 to 3 oxygens (**compounds 4**–**5**; **8**; **10**; **13**–**14**). The molecules of these unidentified clusters were not found in the literature.

**Figure 4:**
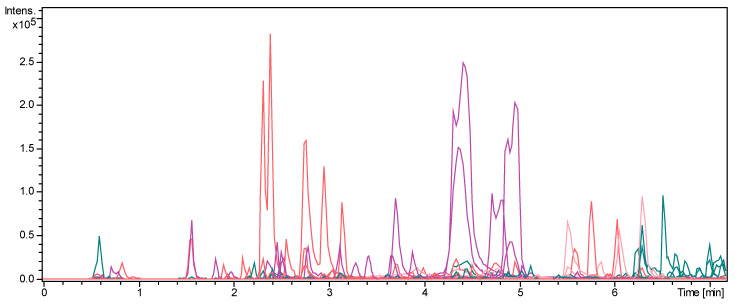
Extract ion chromatograms in negative electrospray ionization of corn silk extract from the characterized molecules. In purple, the compounds consistent with the literature of corn; in red, the characterized molecules in this study; in pink, the molecules with a start of characterization; and in green, the molecules with molecular family information.

Figure 4 presents the wide range of molecules studied in this work. After dereplication of 17 molecules, mainly diglycoside flavones, already described in the literature in the corn silk extract, 24 molecules were identified for the first time in this matrix. Moreover, information about characterization of 8 flavonoids, 6 organic acids and 4 nitrogenous compounds and 29 lipidic compounds were afforded by this study.

## 4. Materials and Methods

### 4.1. Chemicals

Ethanol and acetonitrile were of HPLC analytical grade and were obtained from SDS Carlo Erba (Val-de-Reuil, France). Formic acid was provided by Sigma-Aldrich (Saint Quentin Fallavier, France). Ultrapure water was produced with the PurelabFlex system from Veolia (Wissous, France).

### 4.2. Plant Materials

The sweet corn was cultivated in Martinique. It was then shipped to ICOA in Orléans by air in a polystyrene box containing carbonic ice to maintain a low temperature and then stored at −20 °C until the extraction step.

For the sample, 200 mg of corn silk was weighed and extracted with 10 mL of EtOH 50% using microwave-assisted extraction. The device used was a MicroSYNTH oven (Milestone, Sorisole, Italy) monitored with “easy-control” software. Extraction was performed at a power of 700 W for 3 cycles of 30 s each. The supernatant was recovered and evaporated under nitrogen. The dried extracts were solubilized in the EtOH 50%. Stock solutions were prepared at 10 mg/mL and were stored at 4 °C until use. To have a wide representation of the corn silk composition that could be individual extract-dependent, extractions were carried out on 30 plants and 10 µL of each was mixed. This mixture was analyzed three times.

### 4.3. UHPLC/HRMS/MS

Ultra-high performance liquid chromatography was performed using an Ultimate 3000 RSLC system (Thermo Fisher Scientific Inc., MA, USA) consisting of a binary pump, an online vacuum degasser, an autosampler and a column compartment. Separation of extract was achieved on a Pyramid column (150 mm × 2 mm, 1.8 µm), (Macherey-Nagel, Düren, Germany) fitted with a Nucleodur C18 Gravity (1.8 µm) guard column (Macherey-Nagel, Düren, Germany), kept at 60 °C. Mobile phase A was water containing 0.1% formic acid; mobile phase B was acetonitrile containing 0.08% formic acid. The flow rate was 0.6 mL/min, and the gradient profile was 5 to 20% B in 1.5 min, 20% B for 1.5–2 min, 20 to 35% B in 1.5 min, 35 to 60% B in 1 min, 60% B for 5–6 min, 60 to 100% B in 1.5 min, and 100% B for 1.5 min. The injection volume was 0.6 µL. The equilibration time between two injections was 5 min.

UHPLC was coupled with mass spectrometry detection performed on a maXis UHR-Q-TOF mass spectrometer (Bruker Daltonics, Bremen, Germany). The instrument was used in negative electrospray ionization (ESI-) mode. The capillary voltage was maintained at −4 kV, the gas flow to the nebulizer was set at 2 bar, the drying temperature was heated at 200 °C and the drying gas flow was 10.5 L/min.

Mass spectra were recorded in the data dependent acquisition (DDA) mode with an *m/z* range of 50–1650 for MS spectra and an *m/z* range of 230–660 for MS^2^ spectra. The collision-induced dissociation (CID) energy was applied at 30 eV. Two precursor ions with intensities higher than 1000 au were selected per fragmentation cycle among the most intense ions to be fragmented.

Data were analyzed using Bruker Data Analysis 4.0 software.

### 4.4. Bioinformatic Analysis

After data acquisition, the data analysis Bruker software made it possible to obtain the list of all the *m/z* molecular ions with their retention time. From this list, the Kendrick plot was drawn by applying a mass defect calculation. Kendrick mass is based on the fact that the CH2 group is worth 14 rather than 14.01565 Da [26]. The Kendrick mass defect is calculated from nominal mass minus the mass of this new benchmark. Thus, the molecules which differ only by one or more alkyl groups will have the same mass defect (in this representation), and they will be more easily spotted.

A molecular formula was associated to each ion *m/z* obtained. The list of molecular formulae obtained made it possible to visualize the compounds in the van Krevelen diagram with a representation of the number of hydrogen atoms on the number of carbon atoms as a function of the number of oxygen atoms on the carbon number. The van Krevelen diagram gives another mapping of the corn silk extract. This representation associated with other molecular formulas coming from databases makes it possible to position molecular families and to highlight the most dominant families. [29].

In parallel, the Bruker LC-HRMS/MS data were converted into mzXML format by using MS convert (from the Proteo wizard package), a text-based format used to represent mass spectrometry data describing the scan number, precursor and MS^2^ ion *m/z* and intensity required for the generation of a molecular network (MN). This file was submitted to the GNPS (Global Natural Product Social Molecular Networking) web-based platform to generate an MS-based molecular network [45]. The following parameters were applied to create the molecular network. The mass tolerance for precursor ions was 0.02 Da and for fragment ions was 0.1 Da. The minimum cosine score was 0.6 between the two MS/MS spectra to be connected. The minimum number of common fragment ions between two MS/MS spectra was 2. The nearly identical MS/MS spectra were merged into a single consensus MS/MS spectrum. A node may be connected to up to 10 other nodes. A cluster can have a maximum of 100 nodes. The spectra in the network were then searched against GNPS spectral libraries [45]. The library spectra were filtered in the same manner as the input data.

Once the network was generated, MS2LDA-MotifDB and MolNetEnhancer were generated to highlight the clusters belonging to the same molecular family. MS2LDA is a tool that decomposes molecular fragmentation data [46]. It makes it possible to give information from Mass2Motifs, which are mass fragmentation patterns with fragment peaks and/or neutral losses which often represent molecular substructures. MS^2^ peaks were grouped at 0.01 Da. To converge the LDA model, 1000 iterations were used. The minimum intensity of MS^2^ peaks to include in the MS2LDA analysis was set at 100 au. The number of unsupervised Mass2Motifs MS2LDA was set at 300. MolNetEnhancer is a tool to annotate the network with the chemical classes [47]. This workflow combines the outputs from molecular networking, and here, MS2LDA and chemical classification. A library search was carried out with a minimum of 6 matched peaks, a score threshold at 0.6 and a maximum analog search mass difference of 100. The molecular network was visualized using the Cytoscape software (version 3.7.2). The MN is accessible at the GNPS web site with the following links (MN; MS2LDA; MolNetEnhancer):

https://gnps.ucsd.edu/ProteoSAFe/status.jsp?task=eb3a182150904040aadfc6dff7f135ca (accessed on 7 February 2022)
https://gnps.ucsd.edu/ProteoSAFe/status.jsp?task=38dd1d6c49814e64ba1a6565a75e8b88 (accessed on 7 February 2022)
https://gnps.ucsd.edu/ProteoSAFe/status.jsp?task=5deefe17fed148d49cf194bc2501ddb9 (accessed on 7 February 2022)

## 5. Conclusions

Thus, by combining the information from the Kendrick, van Krevelen and DBE diagrams constructed with the HRMS data of a corn silk hydro-alcoholic extract, 104 molecules were highlighted and described. These compounds consist of 55 flavonoids and 13 organic acids; 29 are lipids and 7 are nitrogenous compounds.

The molecular network obtained with the HRMS/MS data revealed 79 ions which grouped into 5 molecular families. For 24 molecules, experimental MS/MS spectra matched with those of the GNPS library that proposes compound identification. After verifying the proposals with the mass spectra, cross-referencing information from the literature and using the cluster association, 24 other compounds were identified.

Thirty-two phenolic compounds were identified with a structural hypothesis. Among these compounds, about nineteen are flavonoids. With this methodology, the compounds already described in the literature were quickly identified and helped in the identification of unknown compounds. The unknown compounds were described according to their molecular family, then according to their analogous fragmentation pattern. For unknown molecules that could not be identified, information on the basic structure by series of homologs of known compounds was proposed.

Corn silk extract is rich in both molecules of biological interest, such as lipids and flavonoids, and molecules that are involved in the plant’s defense mechanisms, such as *C*-glycosylated flavonoids and benzoxazines. Therefore, better characterization of the phytochemical composition allows better use of the plant.

## Figures and Tables

**Table 1:** Area of molecular families on the van Krevelen diagram [34,35].

	O/C	H/C	N/C
Carbohydrates	≥0.8	≥1.65, <2.7	
Terpenes	≥0.2, ≤0.5	≥0.8, ≤1.7	
Lipids	≤0.6	≥1.32	≤0.126
Phenolic compounds	≥0.3, ≤0.9	≥0.6, ≤1.5	

**Table 2:** Characterization of lipid molecules classified by the homologous carbon series. The background colors indicate the different homologous carbon series.

Compound	Retention Time (min)	*m/z*	Molecular Mass (Da)	Kendric Mass Defect (KMD)	Molecular Formula	H/C	O/C	Double Bond Equivalent (DBE)	Lipid (C: Double Bond + O off Acid)
**1**	6.96	277.1808	278	0.123	C_17_H_26_O_3_	1.53	0.18	5	FA 17:4 + O
**2**	7.00	277.1808	278	0.123	C_17_H_26_O_3_	1.53	0.18	5	FA 17:4 + O
**3**	6.24	293.1754	294	0.146	C_17_H_26_O_4_	1.53	0.24	5	FA 17:4 + 2O
**4**	5.96	309.1725	310	0.167	C_17_H_26_O_5_	1.53	0.29	5	FA 17:4 + 3O
**5**	6.25	309.1703	310	0.169	C_17_H_26_O_5_	1.53	0.29	5	FA 17:4 + 3O
**6**	6.10	311.1862	312	0.155	C_17_H_28_O_5_	1.65	0.29	4	FA 17:3 + 3O
**7**	6.88	291.196	292	0.123	C_18_H_28_O_3_	1.56	0.17	5	FA 18:4 + O
**8**	6.65	307.1911	308	0.146	C_18_H_28_O_4_	1.56	0.22	5	FA 18:4 + 2O
**9**	7.64	293.2117	294	0.11	C_18_H_30_O_3_	1.67	0.17	4	FA 18:3 + O
**10**	6.75	309.2068	310	0.132	C_18_H_30_O_4_	1.67	0.22	4	FA 18:3 + 2O
**11**	5.83	325.2014	326	0.156	C_18_H_30_O_5_	1.67	0.28	4	FA 18:3 + 3O
**12**	6.49	325.2016	326	0.155	C_18_H_30_O_5_	1.67	0.28	4	FA 18:3 + 3O
**13**	6.61	311.2222	312	0.119	C_18_H_32_O_4_	1.78	0.22	3	FA 18:2 + 2O
**14**	6.98	311.2223	312	0.119	C_18_H_32_O_4_	1.78	0.22	3	FA 18:2 + 2O
**15**	5.59	327.2173	328	0.142	C_18_H_32_O_5_	1.78	0.28	3	FA 18:2 + 3O
**16**	6.01	327.2171	328	0.142	C_18_H_32_O_5_	1.78	0.28	3	FA 18:2 + 3O
**17**	6.3	327.2165	328	0.143	C_18_H_32_O_5_	1.78	0.28	3	FA 18:2 + 3O
**18**	6.95	313.2383	314	0.105	C_18_H_34_O_4_	1.89	0.22	2	FA 18:1 + 2O
**19**	5.73	329.2327	330	0.129	C_18_H_34_O_5_	1.89	0.28	2	FA 18:1 + 3O
**20**	6.20	331.2488	332	0.115	C_18_H_36_O_5_	2	0.28	1	FA 18:0 + 3O
**21**	7.57	337.2374	338	0.133	C_20_H_34_O_4_	1.7	0.2	4	FA 20:3 + 2O
**22**	7.83	339.2532	340	0.119	C_20_H_36_O_4_	1.8	0.2	3	FA 20:2 + 2O
**23**	6.72	355.2484	356	0.142	C_20_H_36_O_5_	1.8	0.25	3	FA 20:2 + 3O
**24**	6.42	357.2642	358	0.129	C_20_H_38_O_5_	1.9	0.25	2	FA 20:1 + 3O
**25**	5.91	377.2708	378	0.144	C_23_H_38_O_4_	1.65	0.17	5	FA 23:4 + 2O
**26**	6.08	377.2716	378	0.144	C_23_H_38_O_4_	1.65	0.17	5	FA 23:4 + 2O
**27**	6.30	377.272	378	0.143	C_23_H_38_O_4_	1.65	0.17	5	FA 23:4 + 2O
**28**	6.52	377.272	378	0.143	C_23_H_38_O_4_	1.65	0.17	5	FA 23:4 + 2O
**29**	6.74	377.2722	378	0.143	C_23_H_38_O_4_	1.65	0.17	5	FA 23:4 + 2O

**Table 3:** Characteristics of phenolic compounds. The background colors indicate the different homologous carbon series.

Compound	Retention Time (min)	*m/z*	Molecular Mass (Da)	Kendric Mass Defect (KMD)	Molecular Formula	H/C	O/C	Double Bond Equivalent (DBE)	Description
**1**	3.69	547.1455	548	0.459	C_26_H_28_O_13_	1.077	0.500	13	flavone + 3O digly
**2**	3.99	547.146	548	0.459	C_26_H_28_O_13_	1.077	0.500	13	flavone + 3O digly
**3**	2.77	563.1402	564	0.482	C_26_H_28_O_14_	1.077	0.538	13	flavone + 3O digly * Schaftoside
**4**	5.06	557.1299	558	0.486	C_27_H_26_O_13_	0.963	0.481	15	
**5**	4.79	573.1248	574	0.509	C_27_H_26_O_14_	0.963	0.519	15	
**6**	3.90	589.12	590	0.531	C_27_H_26_O_15_	0.963	0.556	15	flavone + 4O digly
**7**	6.05	589.119	590	0.532	C_27_H_26_O_15_	0.963	0.556	15	flavone + 4O digly
**8**	5.48	605.114	606	0.554	C_27_H_26_O_16_	0.963	0.593	15	
**9**	5.55	621.1088	622	0.579	C_27_H_26_O_17_	0.963	0.629	15	flavone + 4O digly
**10**	5.53	637.104	638	0.601	C_27_H_26_O_18_	0.963	0.667	15	
**11**	4.76	559.1453	560	0.473	C_27_H_28_O_13_	1.037	0.481	14	flavone + 3O digly * apimaysin
**12**	4.25	575.1401	576	0.496	C_27_H_28_O_14_	1.037	0.519	14	flavone + 4O digly * maysin
**13**	4.79	591.1344	592	0.519	C_27_H_28_O_15_	1.037	0.556	14	flavone + 4O digly
**14**	4.34	607.1299	608	0.542	C_27_H_28_O_16_	1.037	0.592	14	flavone + 4O digly
**15**	4.57	561.1614	562	0.459	C_27_H_30_O_13_	1.111	0.481	13	flavone + 3O digly
**16**	3.05	577.1559	578	0.482	C_27_H_30_O_14_	1.111	0.519	13	flavone + 3O digly
**17**	3.11	577.1563	578	0.482	C_27_H_30_O_14_	1.111	0.519	13	flavone + 3O digly
**18**	4.09	577.1552	578	0.483	C_27_H_30_O_14_	1.111	0.519	13	flavone + 3O digly
**19**	2.54	593.1511	594	0.505	C_27_H_30_O_15_	1.111	0.556	13	flavone + 3O digly * vicenin 2
**20**	2.79	593.1511	594	0.505	C_27_H_30_O_15_	1.111	0.556	13	flavone + 3O digly
**21**	3.65	593.1511	594	0.505	C_27_H_30_O_15_	1.111	0.556	13	flavone + 4O digly * luteolin 7-*O*-neohesperidoside
**22**	3.82	593.1511	594	0.505	C_27_H_30_O_15_	1.111	0.556	13	flavone + 4O digly * isoorientin 2″-*O*-rhamnoside
**23**	3.12	609.146	610	0.528	C_27_H_30_O_16_	1.111	0.593	13	flavone + 5O digly * calendoflavobioside
**24**	5.21	587.1399	588	0.509	C_28_H_28_O_14_	1.000	0.5	15	
**25**	4.63	603.135	604	0.532	C_28_H_28_O_15_	1.000	0.536	15	
**26**	6.29	619.13	620	0.555	C_28_H_28_O_16_	1.000	0.571	15	
**27**	3.71	651.1204	652	0.600	C_28_H_28_O_18_	1.000	0.643	15	flavone + 4O digly * luteolin 3′-methylether 7-flucuronosyl-(1–>2)-glucuronide
**28**	4.91	589.1559	590	0.496	C_28_H_30_O_14_	1.071	0.500	14	flavone + 4O digly * 3′-methoxymaysin
**29**	4.96	621.1459	622	0.542	C_28_H_30_O_16_	1.071	0.571	14	flavone + 4O digly
**30**	3.62	637.1414	638	0.564	C_28_H_30_O_17_	1.071	0.607	14	flavone + 4O digly
**31**	3.97	637.1408	638	0.564	C_28_H_30_O_17_	1.071	0.607	14	flavone + 4O digly
**32**	5.06	591.1723	592	0.482	C_28_H_32_O_14_	1.143	0.5	13	flavone + 4O digly * ax-4″-hydroxy-3′-methoxymaysin
**33**	3.84	607.1664	608	0.505	C_28_H_32_O_15_	1.143	0.536	13	flavone + 4O digly * 2″-*O*-L-rhamnosyl-6-*C*-fucosyl-3′-methoxylluteolin
**34**	4.34	607.1663	608	0.506	C_28_H_32_O_15_	1.143	0.536	13	flavone + 4O digly
**35**	2.65	623.1612	624	0.528	C_28_H_32_O_16_	1.143	0.571	13	flavonol + 5O digly * isorhamnetin 3-*O*-neohesperidoside
**36**	5.71	411.0725	412	0.380	C_21_H_16_O_9_	0.762	0.429	14	
**37**	5.42	427.0666	428	0.404	C_21_H_16_O_10_	0.762	0.476	14	
**38**	5.24	429.088	430	0.385	C_21_H_18_O_10_	0.857	0.476	13	flavone + 4O monogly
**39**	4.42	429.0818	430	0.391	C_21_H_18_O_10_	0.857	0.476	13	flavone + 4O monogly
**40**	3.25	431.098	432	0.377	C_21_H_20_O_10_	0.952	0.476	12	flavone + 4O monogly
**41**	4.52	431.0966	432	0.379	C_21_H_20_O_10_	0.952	0.476	12	flavone + 4O monogly
**42**	2.82	447.0942	448	0.399	C_21_H_20_O_11_	0.952	0.524	12	flavone + 4O monogly
**43**	3.86	447.0924	448	0.401	C_21_H_20_O_11_	0.952	0.524	12	flavonol + 3O monogly * kaempferol 3-*O*-glucoside
**44**	3.30	463.0881	464	0.423	C_21_H_20_O_12_	0.952	0.571	12	flavonol + 4O monogly * isoquercitrin
**45**	3.72	449.1087	450	0.387	C_21_H_22_O_11_	1.048	0.524	11	
**46**	2.66	449.1083	450	0.387	C_21_H_22_O_11_	1.048	0.524	11	
**47**	5.79	441.0812	442	0.405	C_22_H_18_O_10_	0.818	0.455	14	
**48**	4.65	475.0868	476	0.438	C_22_H_20_O_12_	0.909	0.545	13	flavone + 4O monogly
**49**	3.89	461.1086	462	0.400	C_22_H_22_O_11_	1.000	0.500	12	flavone + 4O monogly * chrysoeriol *O*-hexoside
**50**	4.12	463.1246	464	0.386	C_22_H_24_O_11_	1.091	0.500	11	flavanone + 4O monogly
**51**	0.62	455.1005	456	0.402	C_23_H_20_O_10_	0.870	0.435	14	
**52**	5.13	457.1135	458	0.391	C_23_H_22_O_10_	0.957	0.435	13	
**53**	5.45	483.0931	484	0.440	C_24_H_20_O_11_	0.833	0.458	15	
**54**	5.61	299.0561	300	0.272	C_16_H_12_O_6_	0.75	0.375	11	flavone + 4O * chrysoeriol
**55**	1.91	329.088	330	0.274	C_14_H_18_O_9_	1.286	0.643	6	
**56**	2.18	339.0721	340	0.300	C_15_H_16_O_9_	1.067	0.600	8	organic acid
**57**	2.23	325.0922	326	0.265	C_15_H_18_O_8_	1.200	0.533	7	organic acid
**58**	2.11	343.1035	344	0.273	C_15_H_20_O_9_	1.333	0.600	6	organic acid * dihydroxyphenylpropanoic acid + hexose
**59**	2.36	337.0926	338	0.278	C_16_H_18_O_8_	1.125	0.500	8	organic acid * quinic acid + coumaric acid
**60**	2.63	337.0924	338	0.278	C_16_H_18_O_8_	1.125	0.500	8	organic acid quinic acid + coumaric acid
**61**	2.94	337.0927	338	0.278	C_16_H_18_O_8_	1.125	0.500	8	organic acid
**62**	2.12	353.0877	354	0.300	C_16_H_18_O_9_	1.125	0.563	8	organic acid * quinic acid + caffeic acid
**63**	2.36	353.0872	354	0.301	C_16_H_18_O_9_	1.125	0.563	8	organic acid * quinic acid + caffeic acid
**64**	2.46	355.1031	356	0.287	C_16_H_20_O_9_	1.250	0.563	7	organic acid
**65**	2.74	367.1029	368	0.301	C_17_H_20_O_9_	1.176	0.529	8	organic acid * feruloylquinic acid
**66**	3.09	367.1033	368	0.300	C_17_H_20_O_9_	1.176	0.529	8	organic acid * feruloylquinic acid
**67**	2.49	405.0797	406	0.366	C_19_H_18_O_10_	0.947	0.526	11	hypothamnolic acid

* represents the molecules described in the literature on *Zea mays*.

**Table 4:** Synthesis of molecules identified in the corn silk extract network.

Family	Compound	Rt (min)	[M-H]^−^	Molecular Formula	Library GNPS	Putative Identification
Flavonoid *O* glycoside	**23**	3.12	609.146	C_27_H_30_O_16_	rutin	Calendoflavobioside *
	**21**	3.65	593.151	C_27_H_30_O_15_	nicotiflorin	luteolin 7-*O*-neohesperidoside *
	**50**	4.12	463.125	C_22_H_24_O_11_		hesperetin *O*-hexoside
	**44**	3.30	463.088	C_21_H_20_O_12_	isoquercitrin	Isoquercitrin *
	**49**	3.89	461.109	C_22_H_22_O_11_		chrysoeriol *O*-hexoside *
	**43**	3.86	447.092	C_21_H_20_O_11_	kaempferol-3-*O*-glucoside	kaempferol-3-*O*-glucoside *
	**55**	1.91	329.088	C_14_H_18_O_9_		glucosyl trihydroxyacetophenone
Flavonoid *C* glycoside		5.53	637.104	C_28_H_30_O_17_		series homologous to methoxymaysin
	**29**	4.96	621.146	C_28_H_30_O_16_		chrysoeriol *O*-deoxyhexose *C*-glucuronide
	**26**	6.29	619.13	C_28_H_28_O_16_		series homologous to luteolin 3′-methylether 7-flucuronosyl-(1–>2)-glucuronide
	**33**	3.84	607.167	C_28_H_32_O_15_		2″-*O*-L-rhamnosyl-6-*C*-fucosyl-3′-methoxylluteolin * diosmetin 8-*C*-(2′’-rhamnosylglucoside)
	**14**	4.34	607.13	C_27_H_28_O_16_		luteolin *O*-deoxyhexose *C*-glucuronide
	**8**	5.48	605.114	C_27_H_26_O_16_		flavone + 4O digly
	**25**	4.63	603.135	C_28_H_28_O_15_		series homologous to luteolin 3′-methylether 7-flucuronosyl-(1–>2)-glucuronide
	**22**	3.82	593.151	C_27_H_30_O_15_	flavone +4O *C*-hex dHex	isoorientin 2″-*O*-rhamnoside
	**19**	2.54	593.151	C_27_H_30_O_15_	MassBank: PR307067 NP-000002(10)	vicenin 2 *
	**32**	5.06	591.178	C_28_H_32_O_14_		ax-4″-hydroxy-3′-methoxymaysin *
	**13**	4.79	591.135	C_27_H_28_O_15_		series homologous to maysin
	**28**	4.91	589.156	C_28_H_30_O_14_		3′-methoxy maysin *
	**6**	3.90	589.12	C_27_H_26_O_15_		flavone + 4O digly
	**7**	6.05	589.119	C_27_H_26_O_15_		flavone + 4O digly
	**24**	5.21	587.14	C_28_H_28_O_14_		series homologous to luteolin 3′-methylether 7-flucuronosyl-(1–>2)-glucuronide
	**18**	4.09	577.158	C_27_H_30_O_14_		apigenin *C*-hexose 2″-*O*-deoxyhexoside
	**12**	4.25	575.14	C_27_H_28_O_14_		maysin *
	**3**	2.77	563.14	C_26_H_28_O_14_	isoschaftoside	schaftoside *
	**11**	4.76	559.146	C_27_H_28_O_13_	5,7-dihydroxy-6-[(2S,3R,4R,6R)-4-hydroxy-6-methyl-5-oxo-3-[(2S,3R,4R,5R,6S)-3,4,5-trihydroxy-6-methyloxan-2-yl]oxyoxan-2-yl]-2-(4-hydroxyphenyl)chromen-4-one	apimaysin *
	**2**	3.99	547.146	C_26_H_28_O_13_		apigenin 6-*C*-deoxyhexose 8-*C*-pentoside *
Organo-oxygen		4.47	502.135	C_24_H_25_NO_11_		
		5.04	486.141	C_24_H_25_NO_10_		
	**67**	2.49	405.08	C_19_H_18_O_10_		hypothamnolic acid
		2.51	372.093	C_15_H_19_NO_10_	DIMBOA + *O*-Hex	DIMBOA-Glucoside *
		1.95	368.099	C_16_H_19_NO_9_		7-hydroxy-2-oxoindole-3-acetic acid 7′-*O*-glucopyranoside *
	**65**	2.74	367.103	C_17_H_20_O_9_	(1R,3R,4S,5R)-1,3,4-trihydroxy-5-[(E)-3-(4-hydroxy-3-methoxyphenyl)prop-2-enoyl]oxycyclohexane-1-carboxylic acid	feruloylquinic acid *
		2.46	356.096	C_15_H_19_NO_9_	HMBOA + *O*-Hex	HMBOA-Glucoside *
	**62**	2.12	353.087	C_16_H_18_O_9_	neochlorogenic acid	chlorogenic acid *
		1.55	344.04	C_10_H_12_N_5_O_7_P	guanosine-3′,5′-cyclic monophosphate (cGMP)	guanosine-3′,5′-cyclic monophosphate (cGMP)
	**58**	2.11	343.103	C_15_H_20_O_9_	3-[3-hydroxy-2-[(2S,3R,4S,5S,6R)-3,4,5-trihydroxy-6-(hydroxymethyl)oxan-2-yl]oxyphenyl]proponoic acid	dihydrocaffeic acid hexoside *
		1.60	342.083	C_14_H_17_NO_9_		DIBOA-Glucoside *
	**56**	2.18	339.072	C_15_H_16_O_9_	6,7-dihydroxycoumarin-glucoside	esculin *
	**60**	2.63	337.093	C_16_H_18_O_8_	(1R,3R,4S,5R)-1,3,4-trihydroxy-5-[(E)-3-(4-hydroxyphenyl)prop-2-enoyl]oxycyclohexane-1-carboxylic acid	coumaroylquinic acid *
		0.85	290.088	C_11_H_17_NO_8_	N-fructosyl pyroglutamate	N-fructosyl pyroglutamate
		0.81	282.084	C_10_H_13_N_5_O_5_	guanosine	guanosine *
Glycero phospholipid		7.25	564.331	C_27_H_52_NO_9_P	LPC 18:2	eicosadienoyl-glycero-(1,2-dihydroxyethoxy)-3-phosphoethanolamine
		7.54	540.33	C_25_H_52_NO_9_P	LPC 16:0	[2-(1,2-dihydroxyethoxy)-3-[2-(dimethylamino)ethoxy-hydroxyphosphoryl]oxypropyl] hexadecanoate
		7.17	476.278	C_23_H_44_NO_7_P	1-(9Z,12Z-octadecadienoyl)-2-hydroxy-sn-glycero-3-phosphoethanolamine	linoleoyl-glycero-phosphoethanolamine
		7.45	452.277	C_21_H_44_NO_7_P	1-palmitoyl-2-hydroxy-sn-glycero-3-phosphoethanolamine	palmitoyl-glycero-phosphoethanolamine
Fatty acid	**24**	6.42	357.264	C_20_H_38_O_5_		FA 20:1 + 3O
	**19**	5.73	329.233	C_18_H_34_O_5_	FA 18:1 + 3O	9,12,13-triHOME *
	**15**	5.86	327.217	C_18_H_32_O_5_	(10E,15E)-9,12,13-trihydroxyoctadeca-10,15-dienoic acid	9,12,13-triHODE * FA 18:2 + 3O
	**11**	5.83	325.202	C_18_H_30_O_5_		FA 18:3 + 3O

* represents the molecules described in the literature on *Zea mays*.

## Data Availability

The GNPS-generated LC-MS/MS network can be accessed at: https://gnps.ucsd.edu/ProteoSAFe/status.jsp?task=5deefe17fed148d49cf194bc2501ddb9, accessed on 7 February 2022.
